# Comparative Analysis of Mechanical Variables in Different Exercises Performed with a Rotational Inertial Device in Professional Soccer Players: A Pilot Study

**DOI:** 10.3390/jfmk10030279

**Published:** 2025-07-18

**Authors:** Álvaro Murillo-Ortiz, Luis Manuel Martínez-Aranda, Moisés Falces-Prieto, Samuel López-Mariscal, Francisco Javier Iglesias-García, Javier Raya-González

**Affiliations:** 1 Research Group on Sport and Physical Education for Personal and Social Development (GIDEPSO), Department of Specific Didactics, Faculty of Education Sciences and Psychology, University of Córdoba, 14004 Córdoba, Spain; alvaromurillo97@gmail.com (Á.M.-O.); rayagonzalezjavier@gmail.com (J.R.-G.); 2 Department of Sports and Computer Sciences, Faculty of Sports Sciences, Universidad Pablo de Olavide, 41013 Seville, Spain; samuellopm@gmail.com; 3 Science-Based Training Research Group (SEJ-680), Physical Performance and Sports Research Center, Universidad Pablo de Olavide, 41013 Seville, Spain; 4 GIR07, Research Group in Physical Activity and Sports Sciences, University Isabel I, 09001 Burgos, Spain; mfalpri@gmail.com; 5 High Performance Department KMSK Deinze, 9800 Deinze, Belgium; javi.iglesias30@gmail.com; 6 Research Group CTS563, Faculty of Education, Málaga University, 29071 Málaga, Spain

**Keywords:** assessment, strength, flywheel, kinetics, performance, symmetry

## Abstract

**Background:** Soccer performance is largely dependent on high-intensity, unilateral actions such as sprints, jumps, and changes of direction. These demands can lead to strength and power differences between limbs, highlighting the importance of individualised assessment in professional players. Rotational inertial devices offer a valuable method to evaluate and train these mechanical variables separately for each leg. The aim of this study was twofold: (a) to characterise the mechanical variables derived from several lower-body strength exercises performed on rotational inertial devices, all targeting the same muscle group; and (b) to compare the mechanical variables between the dominant and non-dominant leg for each exercise. **Methods:** Twenty-six male professional soccer players (age = 26.3 ± 5.1 years; height = 182.3 ± 0.6 cm; weight = 75.9 ± 5.9 kg; body mass index = 22.8 ± 1.1 kg/m^2^; fat mass percentage = 9.1 ± 0.6%; fat-free mass = 68.8 ± 5.3 kg), all belonging to the same professional Belgian team, voluntarily participated in this study. The players completed a single assessment session consisting of six unilateral exercises (i.e., quadriceps hip, hamstring knee, adductor, quadriceps knee, hamstring hip, and abductor). For each exercise, they performed two sets of eight repetitions with each leg (i.e., dominant and non-dominant) in a randomised order. **Results:** The quadriceps hip exercise resulted in higher mechanical values compared to the quadriceps knee exercise in both limbs (*p* < 0.004). Similarly, the hamstring hip exercise produced greater values across all variables and limbs (*p* < 0.004), except for peak force, where the hamstring knee exercise exhibited higher values (*p* < 0.004). The adductor exercise showed higher peak force values for the dominant limb (*p* < 0.004). The between-limb comparison revealed differences only in the abductor exercise (*p* < 0.004). **Conclusions:** These findings suggest the necessity of prioritising movement selection based on targeted outcomes, although it should be considered that the differences between limb differences are very limited.

## 1. Introduction

Modern soccer is a team sport in which players must adequately cope with high demands to perform effectively and reduce the risk of injury [1]. Specifically, these demands are mainly related to high-intensity actions such as jumps, sprints, and changes of direction [2]. Due to the specific nature of soccer, these actions are executed unilaterally, which impacts the strength and power levels of each limb [3]. Consequently, it is necessary to consider these differences when designing training programmes, in order to optimise their effects and, in turn, enhance the level of preparation of soccer players [4].

Given this, evaluating each limb separately is crucial for detecting strength differences in athletes [5]. Previous studies have shown that soccer players often exhibit differences between their dominant and non-dominant legs. For instance, a study on young soccer players found significant differences in muscle strength between the dominant and non-dominant leg, supporting the importance of assessing each limb individually [6]. Similarly, a study on lower-limb strength imbalance in professional soccer players found that strength differences between legs can influence performance and injury risk [7]. Furthermore, studies comparing the right leg to the left leg have observed similar differences. Research on intra- and inter-limb strength imbalances in soccer players also found significant differences between legs, emphasising the need for individualised assessments [8]. In soccer players, the existence of a skilled leg (e.g., for passing and striking) and a non-skilled leg (e.g., for support functions) must be considered [6]. In this regard, performing such markedly different functions could influence the characteristics of each limb, making an analysis of strength and power differences following this classification essential, mainly including exercises focused on quadriceps, hamstring, adductors and abductors, and differentiating according to the joint involved (i.e., hip vs. knee).

Understanding the specific outcomes derived from the concentric and eccentric phases of movement in soccer is crucial for gaining a more comprehensive knowledge of players’ needs. In this context, rotational inertial devices appear to be an appropriate tool, as they allow for the individual assessment of each limb while considering both movement phases. In addition, these devices offer resistance tailored to each individual according to their maximal voluntary contraction [9]. In a previous study, Raya-González et al. [10] analysed power production using a rotational inertial device, comparing concentric (CON) and eccentric (ECC) phases in the dominant and non-dominant leg. The authors observed that mean power was higher during the concentric phase compared to the eccentric phase, while eccentric overload was only partially achieved, with higher eccentric than concentric peak power observed only in the dominant leg during unilateral lateral exercise. Furthermore, the non-dominant leg presented slightly higher values across all the assessed power variables, although only eccentric mean power and concentric peak power showed significant differences. Similarly, Raya-González [11] reported differences in power production between the two legs during a rotational inertial device exercise. Specifically, higher power values were observed in the non-dominant leg, although significant differences were recorded only for eccentric mean power and concentric peak power. Nevertheless, previous studies have mainly compared the weak leg versus the strong leg or have focused on a single exercise (i.e., half squat). Therefore, it appears necessary to understand the demands of the most common exercises performed on rotational inertial devices (i.e., focused on quadriceps, hamstring, adductor, and abductor muscle groups). In addition, Murillo-Ortiz et al. [12] aimed to establish limb-specific normative values for flywheel-derived power indices in professional soccer players, while exploring the relationships between power indices across variables. This investigation revealed significant correlations between indices in all power variables, with the Qhip:Qknee and Hhip:Hknee concentric ratios emerging as the most clinically actionable biomarkers for rapid screening. Also, it is relevant to analyse several exercises focused on the same muscle group, which could be relevant to better exercise selection based on desired outcomes [13].

The aim of this study was twofold: (a) to compare mechanical variables derived from different exercises targeting the same muscle group; and (b) to compare the mechanical variables between the dominant and non-dominant leg for each exercise in male professional soccer players. Based on previous studies [10], we hypothesised that greater values across all power and strength variables would be found in all exercises when performed with the dominant leg.

## 2. Materials and Methods

### 2.1. Study Design

A cross-sectional design was applied to analyse the mechanical variables derived from several lower-body strength exercises performed on rotational inertial devices, comparing exercises focused on the same muscle group and comparing the dominant and non-dominant leg for each exercise. Players completed a single assessment session at the club’s facilities (17–22 °C, 60–70% relative humidity) in the morning, between 9 a.m. and 11 a.m. This session comprised six exercises (i.e., two sets of eight repetitions): quadriceps hip (Qhip), hamstring knee (Hknee), adductor (ADD), quadriceps knee (Qknee), hamstring hip (Hhip), and abductor (ABD), completed in a randomised order. The dominant leg was defined as the leg the soccer player primarily prefers to use when kicking the ball, while the non-dominant leg was defined as the leg used for support when kicking [10]. All exercises were performed randomly with both legs. Prior to the assessment, players completed a standardised warm-up, including cycling, joint mobility exercises, dynamic stretching, bilateral and unilateral jumps, and a submaximal set using rotational inertial devices. The assessment session was supervised by the strength and conditioning staff, who provided verbal encouragement during the exercises. Players were instructed to maintain their usual habits, ensure optimal hydration and carbohydrate intake during the 24 h prior to the assessment, and to avoid consuming caffeinated beverages in the morning.

### 2.2. Participants

Twenty-six male professional soccer players (age = 26.3 ± 5.1 years; height = 182.3 ± 0.6 cm; weight = 75.9 ± 5.9 kg; body mass index = 22.8 ± 1.1 kg/m^2^; fat mass percentage = 9.1 ± 0.6%; fat-free mass = 68.8 ± 5.3 kg) voluntarily participated in this study. All players belonged to the same professional team, which competed in the second division of the Belgian league (i.e., Challenger Pro League). Using GPower software (version 3.1.9.2, Universität Kiel, Germany), an a priori power calculation determined that a sample size of at least 20 participants was necessary to identify a large effect size (ES = 0.90) with a power of 84% (β = 0.16) and a significance threshold of α = 0.05. To be included in the analysis, players were required to have fully completed the assessment sessions (i.e., two sets for all exercises), to be free from partial or chronic injuries (e.g., muscle–tendon or joint injuries), and to have accumulated a minimum of approximately five years of licensed soccer playing experience. Participants were informed prior to the start of the investigation about the procedures, aims, and risks, and all provided their informed consent. In addition, the study protocol adhered to the principles of the Declaration of Helsinki and was approved by the Ethics Committee of the University of Córdoba (Code: CEIH-24-50; date: 20 November 2024).

### 2.3. Procedures

**Figure 1:**
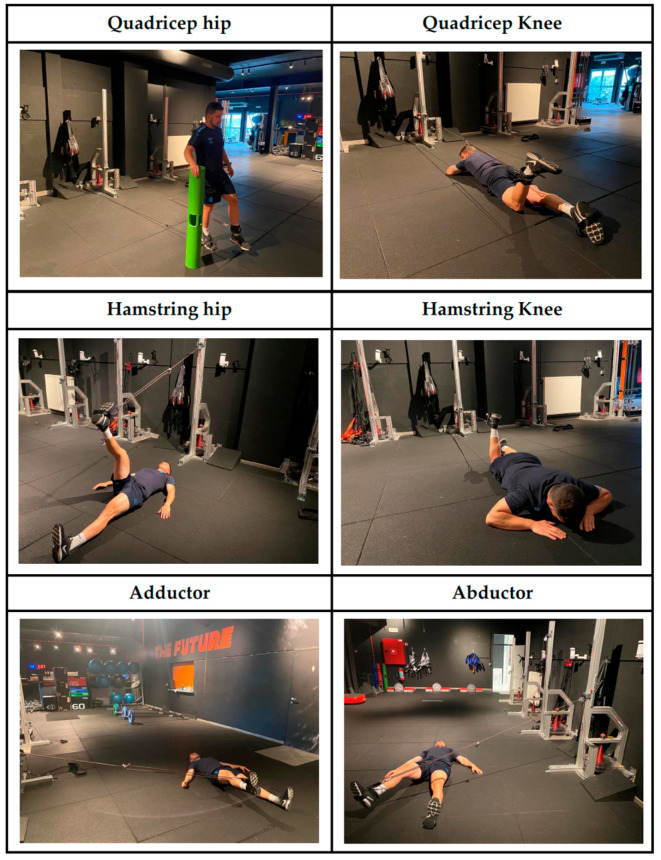
Exercises by muscle group.

Players completed a single assessment session to evaluate their mechanical variables during several lower-body strength exercises performed on rotational inertial devices. Six exercises were included in the assessment: Qhip, Qknee, Hhip, Hknee, ADD, and ABD (Figure 1). For a better understanding, an explanation for each exercise is presented below:

- *Qhip:* Participants started in a standing position with hips in neutral alignment, knees slightly flexed at 10°, and feet shoulder-width apart. Then, they initiated the unilateral hip extension to 10° hyperextension against inertial resistance, to then initiate a controlled eccentric return to the initial posture.
- *Qknee:* Players lay prone with hips in neutral alignment, knees actively flexed to 90°, and ankles maintained at 0° dorsiflexion. Then, they performed an unilateral knee extension to actively extend the knee joint from 90° flexion to full extension. When players reach the final position, they must break the return movement until they reach the initial position.
- *Hhip:* Participants lay supine with hips flexed at 60°, knees fully extended, and ankles secured in foot pads attached to a rotational inertia device; then, players performed a unilateral hamstring curl by simultaneously extending the hip from 60° flexion to neutral against rotational inertial resistance, initiating a controlled eccentric return to the initial posture.
- *Hknee:* Soccer players lay prone with hips in neutral alignment, knees fully extended, and ankles secured to a rotational inertia device; then, players performed a unilateral knee-dominant hamstring curl by flexing the knee joint from 0° to 120° flexion against rotational inertial resistance, initiating a controlled eccentric return to the initial posture.
- *ADD:* Participants lay supine with hips in neutral alignment (0° flexion/extension) and thighs abducted to 30°, knees extended, while secured to a rotational inertia device via ankle pads; then, players performed unilateral hip adduction by driving the thigh from 30° abduction to neutral (0° adduction) against rotational inertial resistance, initiating a controlled eccentric return to the initial posture.
- *ABD:* Players lay supine with hips in neutral alignment, thighs adducted to 10°, and knees extended while secured to a rotational inertia device via ankle pads; then, players performed unilateral hip abduction by driving the thigh from 10° adduction to 45° abduction against rotational inertial resistance, initiating a controlled eccentric return to the initial posture.

Each player performed two randomly ordered sets of ten repetitions for each exercise (inertia: 0.0335 kg·m^−2^), with the first two repetitions discarded to allow for cone acceleration. A four-minute rest period was provided between sets. During each repetition, players were instructed to execute the concentric phase as rapidly as possible and to delay the braking action until the final third of the eccentric phase [14]. All exercises were performed on the Pulley Pro C3 device (Proinertial^®^, Barcelona, Spain), which was connected to a rotary encoder (Chronojump, Barcelona, Spain). The encoder enabled the export of mechanical data through its dedicated software (Chronojump, Barcelona, Spain, v2.3.0-2423). Both the rotational inertial device and the encoder have been previously used for investigation purposes [15,16]. In this regard, Mak, Bishop and Beato [17], showed that flywheel testing is reliable (ICC = 0.66–0.99, r = 0.69–0.97, α = 0.85–0.98) and valid for the athletic population when subjects undergo two familiarization sessions, which were performed prior to the assessment. Mechanical variables, including mean power, peak power, mean velocity, peak velocity, mean force, and peak force, were recorded for both the concentric (CON) and eccentric (ECC) phases of each exercise. For subsequent statistical analysis, the repetition with the highest mean power in the concentric phase, taken from the second set, was selected. To ensure proper technique, all exercises were supervised by a strength and conditioning coach, and a mirror was placed in front of the participants to provide visual feedback [18].

### 2.4. Statistical Analysis

Results are presented as means ± standard deviations (SD). Normal distribution and homogeneity of variance were confirmed for all dependent variables using the Shapiro–Wilk and Levene tests. A paired-samples *t*-test was used to compare the mechanical variables related to exercises targeting the same muscle group for the dominant and non-dominant legs; the same test was also applied to compare the mechanical variables between the dominant and non-dominant leg for each individual exercise. Effect sizes (ESs) were calculated using Cohen’s ES to assess the magnitude of the effects. ESs were interpreted as follows: <0.2, trivial; 0.20 to 0.49, small; 0.50 to 0.80, moderate; and >0.80, large [19]. Data analysis was carried out using Jeffrey’s Amazing Statistics Program (JASP 0.18.1; The JASP Team, Amsterdam, The Netherlands). To control for Type I error due to multiple *t*-tests, a Bonferroni correction was applied, and consequently, statistical significance was set at *p* < 0.004.

## 3. Results

Table 1, Table 2 and Table 3 present the descriptive values and comparisons between exercises targeting the same muscle group.

### 3.1. Quadriceps Exercises

Significant differences in favour of the Qhip exercise compared to the Qknee exercise were observed across all the variables (*p* < 0.01), except for peak power ECC and peak force ECC in the dominant leg. Similar results were found in the non-dominant leg (*p* < 0.001), where no significant differences were observed for the peak force ECC variable.

### 3.2. Hamstring Exercises

Significantly greater values in favour of the Hhip exercise were found in both dominant and non-dominant limbs (*p* < 0.01) across all the variables, except for peak power ECC in dominant limbs. Higher values of peak force CON and peak force ECC were shown for Hknee.

### 3.3. Adductor vs. Abductor Exercises

For the comparison between ADD and ABD exercises, no significant differences were found in the dominant limb for the peak force variable (CON and ECC), in favour of the ADD exercise.

Between-limb comparisons are shown in Table 4. Significant differences between dominant and non-dominant limbs were found only for the ABD exercise, with greater values for the non-dominant limb in mean velocity ECC and peak force CON.

## 4. Discussion

This study aimed to compare mechanical variables derived from different exercises targeting the same muscle group. Additionally, it sought to compare the mechanical variables between the dominant and non-dominant leg for each exercise in male professional soccer players. This is the first study to perform such a comparison, as previous research has primarily contrasted strong versus weak limbs and often focused on a single exercise (e.g., half-squat). Overall, the Qhip exercise resulted in higher mechanical values compared to the Qknee exercise in both the dominant and non-dominant limbs. Similarly, the Hhip exercise produced greater values across all variables and limbs, except for peak force (CON and ECC), where the Hknee exercise exhibited higher values. Regarding the ADD-ABD comparison, peak force (CON and ECC) was higher in the ADD exercise for the dominant limb. Finally, the between-limb comparison revealed significant differences only in the ABD exercise, with the non-dominant limb demonstrating higher values in mean velocity (ECC) and peak force (CON).

A critical component of exercise prescription lies in systematically comparing movements that target the same muscle groups yet differ in kinematic patterns, neuromuscular demands, and biomechanical outputs [20]. For instance, although both the Qhip and Qknee exercises are designed to activate the quadriceps group, empirical data reveal significant disparities in strength and power outcomes, with the Qhip exercise demonstrating superior performance metrics (ES = from 0.35 to 1.52). This divergence may be attributed to its capacity to exploit a larger range of motion at the hip joint [21], thereby facilitating greater force production through enhanced stretch–shortening cycle engagement [22]. Additionally, the integration of synergistic musculature, notably the gluteus maximus, which contributes to hip extension torque and pelvic stabilisation [23], may further amplify force transmission during Qhip execution [9,24]. Crucially, these inter-exercise discrepancies persist bilaterally, as observed in both dominant and non-dominant limbs, underscoring the primacy of task-specific mechanics over limb dominance in determining neuromuscular adaptations [25]. Such insights hold profound practical implications: exercise selection must be contextualised not only within performance paradigms (e.g., maximising power output in athletes) but also in clinical settings, where modulating mechanical stress on specific tissues is paramount for injury mitigation (e.g., reducing patellofemoral load during rehabilitation) or addressing asymmetries [5]. Further evidence of movement-specific adaptation emerges from the comparative analysis of adductor and abductor exercises. Notably, the dominant limb exhibited greater peak force in adduction tasks (ES = from 0.62 to 0.70), whereas the non-dominant limb demonstrated higher peak velocity during abduction (ES = from 0.27 to 0.51). This compensatory phenomenon may reflect sport-specific neuromuscular patterning, particularly in soccer athletes, where repetitive lateral stabilisation, unilateral deceleration, and multiplanar direction changes impose heterogeneous demands on the adductor–abductor complex [26]. For example, the dominant leg’s adductor strength may facilitate forceful kicking mechanics, while the non-dominant leg’s abductor velocity could enhance dynamic stability during cutting manoeuvres, potentially predisposing athletes to asymmetrical overload injuries if unaddressed [8,27]. These findings collectively advocate for a nuanced, context-driven approach to exercise programming, one that prioritises biomechanical specificity alongside individual functional demands.

Assessing inter-limb differences between the dominant and non-dominant leg holds significant scientific and practical relevance, particularly in soccer, a sport characterised by pronounced functional specialisation of the lower extremities [28]. The dominant leg typically assumes dynamic, high-force roles such as ball striking and precision passing, whereas the non-dominant leg predominantly facilitates stabilisation, weight-bearing, and directional control during unilateral movements [6]. Despite these distinct kinematic responsibilities, the current study revealed no significant differences in force production or neuromuscular activation during isolated quadriceps and hamstring exercises. This suggests that functional laterality, while critical for sport-specific skills, may not inherently translate into mechanical dissimilarities in these muscle groups when assessed under controlled, non-task-specific conditions [25]. Such findings challenge conventional assumptions about limb dominance, advocating instead for a paradigm shift toward strength-based stratification (i.e., strong versus weak leg) to optimise training specificity and address individual asymmetries, irrespective of innate laterality [29]. Notably, the adductor musculature exhibited bilateral symmetry in performance metrics, likely attributable to its consistent role in maintaining pelvic stability and controlling frontal-plane motion during multidirectional tasks, a demand equally imposed on both limbs during soccer-specific actions such as cutting, lateral shuffling, or abrupt decelerations [26]. In contrast, the abductor muscles demonstrated elevated force–velocity profiles in the non-dominant leg, a phenomenon potentially linked to its compensatory stabilisation requirements during high-velocity striking actions [26]. Specifically, the non-dominant abductor complex may act as a critical modulator of hip and pelvic alignment, counteracting rotational and lateral shear forces generated during kicking or sudden directional changes [30]. These differences underscore the interplay between sport-specific neuromuscular adaptation and inherent biomechanical demands, wherein the non-dominant limb develops actions that preserve dynamic equilibrium under load.

### Limitations and Future Research Lines

This study presents several limitations that warrant consideration. First, the experimental design employed a single assessment session, precluding longitudinal analysis of chronic neuromuscular adaptations or transient performance fluctuations associated with cumulative fatigue. Such a cross-sectional approach limits insight into temporal variations in strength–power expression, which may evolve differentially across training cycles or recovery phases. Second, although the exercise order was randomised between limbs to mitigate acute carryover effects, the potential influence of residual neuromuscular fatigue or potentiation, particularly given the high-intensity nature of the tasks, cannot be entirely discounted. Third, confounding variables such as recent injury history (<12 months) and positional demands (e.g., defender versus forward roles) were not systematically controlled. These factors are known to modulate force-generation capacity and movement efficiency in soccer athletes, as positional specificity alters exposure to distinct loading patterns (e.g., frequent accelerations in forwards versus decelerations in defenders), potentially engendering task-dependent neuromuscular adaptations. Finally, the study was conducted in a single team and in a specific temporal moment, so the generalization of the obtained results should be undertaken with caution.

Future studies should prioritise classifying limbs by strength thresholds (e.g., >10% asymmetry) over functional dominance to address inter-limb differences more effectively. Incorporating surface electromyography could disentangle neural versus peripheral contributions to force discrepancies, while kinematic analyses of multi-joint movements (e.g., hip–knee coordination) may reveal compensatory limb strategies. Additionally, integrating sport-specific tasks (e.g., fatigued kicking, unanticipated cuts) would enhance ecological validity.

## 5. Conclusions

The present findings demonstrate distinct exercise-specific and limb-dependent mechanical adaptations during lower-body resistance training. Notably, the Qhip exercise consistently elicited superior mechanical outputs (e.g., force, velocity) compared to the Qknee exercise across both limbs. Similarly, the Hhip exercise outperformed the Hknee exercise in most parameters, except for peak force. Inter-limb differences were found only in the ABD exercise, with the non-dominant limb exhibiting greater eccentric power, velocity, and concentric force. On the other hand, it is essential to highlight that no inter-limb differences were observed in any exercise, with the exception of the abductor exercise. Regarding this, practitioners should choose hip-dominant exercises for maximum power, and it is recommended to screen adductor asymmetries weekly.

### Practical Applications

The observed exercise-specific and limb-dependent mechanical profiles provide actionable insights for strength and conditioning programming in professional soccer populations. Practitioners should prioritise hip-dominant exercises (i.e., Qhip and Hhip) when aiming to maximise force–velocity outputs during lower-body training, particularly for power development or sports requiring explosive hip extension (e.g., sprinting, jumping). However, the preserved peak force in the Hknee exercise suggests its retained utility in contexts emphasising isolated knee flexion strength, such as hamstring injury rehabilitation. Moreover, incorporating this information into rehabilitation protocols may assist in selecting exercises with lower strength and power requirements early in the process, progressively integrating those with greater demands, while carefully considering the specific nature of each injury. The pronounced non-dominant limb superiority in ABD exercises underscores the need to incorporate targeted abductor training (e.g., resisted lateral band walks, single-leg lateral hops) to enhance dynamic stabilisation during high-velocity directional changes, a critical demand in multidirectional sports like soccer.

For holistic athletic development, programmes should combine hip-driven compound movements with limb-stratified exercises, adjusting loading parameters (e.g., velocity emphasis for the non-dominant limb, force emphasis for the dominant limb) to address inherent asymmetries. Also, the absence of inter-limb differences must be considered when designing unilateral training programs based on rotational inertia devices, suggesting the need for individualized assessments for each player. Regular monitoring of inter-limb differences via force–velocity profiling is recommended to individualise training loads and mitigate injury risks associated with neuromuscular imbalances.

## Figures and Tables

**Table 1:** Descriptive values and comparison between quadricep exercises.

Variable	Exercise			
	Dom		Ndom	
	Quadricep Hip	Quadricep Knee	Quadricep Hip	Quadricep Knee
Mean power CON (W)	312.64 ± 94.06 ***	205.24 ± 58.83	311.55 ± 93.27 ***	203.06 ± 54.55
Mean power ECC (W)	309.43 ± 100.681 ***	200.62 ± 61.27	305.47 ± 97.46 ***	198.04 ± 57.20
Peak power CON (W)	522.12 ± 156.84 ***	376.29 ± 123.06	506.56 ± 156.83 ***	366.96 ± 88.01
Peak power ECC (W)	616.72 ± 250.52	529.52 ± 178.35	633.53 ± 294.31	529.52 ± 178.35
Mean velocity CON (m/s)	2.63 ± 0.33 ***	2.02 ± 0.25	2.61 ± 0.31 ***	2.02 ± 0.17
Mean velocity ECC (m/s)	2.88 ± 0.46 ***	2.18 ± 0.31	2.88 ± 0.46 ***	2.20 ± 0.29
Peak velocity CON (m/s)	4.14 ± 0.47 ***	3.23 ± 0.38	4.15 ± 0.45 ***	3.23 ± 0.34
Peak velocity ECC (m/s)	4.13 ± 0.50 ***	3.19 ± 0.37	4.15 ± 0.462 ***	3.21 ± 0.34
Mean force CON (N)	138.31 ± 26.67 ***	114.76 ± 22.48	138.55 ± 26.20 ***	114.86 ± 21.41
Mean force ECC (N)	137.21 ± 29.79 ***	114.06 ± 24.71	134.72 ± 27.51 ***	112.38 ± 23.30
Peak force CON (N)	247.96 ± 53.12 ***	197.04 ± 51.20	255.44 ± 65.65 ***	192.64 ± 37.09
Peak force ECC (N)	344.33 ± 123.77	286.03 ± 84.52	337.55 ± 129.62	295.13 ± 130.58

Abbreviations: Concentric (CON); Eccentric (ECC); Dominant (Dom); Non-Dominant (Ndom). *** *p* < 0.004.

**Table 2:** Descriptive values and comparison between hamstring exercises.

Variable	Exercise			
	Dom		Ndom	
	Hamstring Hip	Hamstring Knee	Hamstring Hip	Hamstring Knee
Mean power CON (W)	259.46 ± 58.60 ***	109.19 ± 30.44	249.87 ± 51.21 ***	107.43 ± 33.18
Mean power ECC (W)	262.96 ± 59.25 ***	111.90 ± 35.39	255.96 ± 56.56 ***	112.24 ± 37.16
Peak power CON (W)	453.17 ± 92.45 ***	216.83 ± 81.75	435.71 ± 90.91 ***	204.04 ± 47.45
Peak power ECC (W)	488.18 ± 135.51 ***	352.37 ± 155.40	492.36 ± 139.76	402.62 ± 178.21
Mean velocity CON (m/s)	2.50 ± 0.22 ***	1.61 ± 0.21	2.49 ± 0.18 ***	1.58 ± 0.22
Mean velocity ECC (m/s)	2.73 ± 0.28 ***	1.88 ± 0.25	2.70 ± 0.27 ***	1.90 ± 0.25
Peak velocity CON (m/s)	4.43 ± 0.40 ***	2.37 ± 0.25	4.38 ± 0.34 ***	2.36 ± 0.28
Peak velocity ECC (m/s)	4.36 ± 0.41 ***	2.38 ± 0.26	4.32 ± 0.37 ***	2.37 ± 0.27
Mean force CON (N)	110.27 ± 15.91 ***	82.70 ± 15.47	107.43 ± 15.05 ***	82.16 ± 16.72
Mean force ECC (N)	113.33 ± 15.89 ***	82.44 ± 18.74	111.88 ± 16.47 ***	82.51 ± 18.31
Peak force CON (N)	158.69 ± 39.44 ***	205.80 ± 62.51	151.64 ± 24.04 ***	194.77 ± 39.81
Peak force ECC (N)	203.29 ± 50.90 ***	330.04 ± 98.04	197.60 ± 46.11 ***	362.28 ± 114.36

Abbreviations: Concentric (CON); Eccentric (ECC); Dominant (Dom); Non-Dominant (Ndom). *** *p* < 0.004.

**Table 3:** Descriptive values and comparison between adductor–abductor exercises.

Variable	Exercise			
	Dom		Ndom	
	Adductor	Abductor	Adductor	Abductor
Mean power CON (W)	2.43 ± 41.78	119.94 ± 29.60	117.26 ± 34.63	125.66 ± 31.92
Mean power ECC (W)	123.16 ± 44.00	116.49 ± 26.83	119.25 ± 37.75	123.55 ± 30.49
Peak power CON (W)	202.91 ± 60.22	200.18 ± 44.29	201.01 ± 50.19	203.31 ± 51.73
Peak power ECC (W)	233.64 ± 98.30	224.66 ± 74.34	218.70 ± 71.10	242.28 ± 75.23
Mean velocity CON (m/s)	1.95 ± 0.23	1.91 ± 0.23	1.93 ± 0.21	2.01 ± 0.26
Mean velocity ECC (m/s)	2.10 ± 0.25	2.02 ± 0.26	2.07 ± 0.26	2.13 ± 0.27
Peak velocity CON (m/s)	3.14 ± 0.38	3.21 ± 0.36	3.19 ± 0.36	3.29 ± 0.37
Peak velocity ECC (m/s)	3.12 ± 0.38	3.17 ± 0.35	3.07 ± 0.36	3.26 ± 0.37
Mean force CON (N)	72.55 ± 15.17	70.06 ± 10.09	69.93 ± 12.80	71.67 ± 10.91
Mean force ECC (N)	73.14 ± 17.16	69.12 ± 8.69	71.99 ± 14.34	71.11 ± 10.41
Peak force CON (N)	128.80 ± 34.08 ***	104.82 ± 18.86	126.12 ± 31.19	117.29 ± 27.91
Peak force ECC (N)	162.85 ± 56.79 ***	127.85 ± 29.93	157.80 ± 48.70	142.16 ± 39.12

Abbreviations: Concentric (CON); Eccentric (ECC); Dominant (Dom); Non-dominant (Ndom). *** *p* < 0.004.

**Table 4:** Between-limbs comparison (i.e., dominant vs. non-dominant).

Variable	Exercise					
	Quadricep Hip (*p*; ES)	Quadricep Knee (*p*; ES)	Hamstring Hip (*p*; ES)	Hamstring Knee (*p*; ES)	Adductor (*p*; ES)	Abductor (*p*; ES)
Mean power CON (W)	0.906; 0.023	0.702; 0.076	0.149; 0.292	0.492; 0.137	0.290; 0.212	0.076; 0.364
Mean power ECC (W)	0.624; 0.097	0.729; 0.069	0.360; 0.183	0.901; 0.025	0.467; 0.145	0.040; 0.425
Peak power CON (W)	0.448; 0.151	0.609; 0.102	0.235; 0.239	0.458; 0.148	0.823; 0.044	0.659; 0.087
Peak power ECC (W)	0.392; 0.171	0.737; 0.067	0.871; 0.032	0.076; 0.363	0.228; 0.243	0.192; 0.263
Mean velocity CON (m/s)	0.542; 0.121	0.956; 0.011	0.703; 0.076	0.327; 0.196	0.447; 0.152	0.012; 0.533
Mean velocity ECC (m/s)	0.955; 0.011	0.627; 0.097	0.436; 0.155	0.474; 0.143	0.340; 0.191	0.001; 0.710
Peak velocity CON (m/s)	0.882; 0.029	0.965; 0.009	0.370; 0.179	0.517; 0.129	0.497; 0.135	0.047; 0.410
Peak velocity ECC (m/s)	0.732; 0.068	0.761; 0.060	0.512; 0.130	0.598; 0.105	0.332; 0.194	0.036; 0.436
Mean force CON (N)	0.935; 0.016	0.962; 0.009	0.103; 0.332	0.697; 0.077	0.158; 0.285	0.116; 0.319
Mean force ECC (N)	0.351; 0.186	0.571; 0.113	0.496; 0.135	0.957; 0.011	0.582; 0.109	0.091; 0.345
Peak force CON (N)	0.310; 0.203	0.489; 0.138	0.220; 0.247	0.410; 0.164	0.650; 0.090	0.002; 0.685
Peak force ECC (N)	0.440; 0.154	0.604; 0.103	0.481; 0.140	0.113; 0.322	0.496; 0.136	0.011; 0.537

Abbreviations: Concentric (CON); Eccentric (ECC).

## Data Availability

The data are available upon request to the corresponding author.

## Abbreviations

The following abbreviations are used in this manuscript:

Qhip	Quadriceps hip
Hhip	Hamstring hip
Qknee	Quadriceps knee
Hknee	Hamstring knee
ABD	Abduction
ADD	Adduction
CON	Concentric
ECC	Eccentric
